# Anti-proliferative effect of LXR agonist T0901317 in ovarian carcinoma cells

**DOI:** 10.1186/1757-2215-3-13

**Published:** 2010-05-26

**Authors:** James J Rough, M Alexandra Monroy, Smitha Yerrum, John M Daly

**Affiliations:** 1 Department of Surgery, Temple University School of Medicine, Philadelphia, PA, USA; 2 Department of Anatomy and Cell Biology, Temple University School of Medicine, Philadelphia, PA, USA

## Abstract

**Background:**

Ovarian cancer is the most common cause of cancer related death from gynecologic tumors in the United States. The insidious nature of the disease precludes early diagnosis, therefore surgical debulking and chemotherapy are considered as standard treatment modalities for advanced stages. We investigated the effect of the LXR agonist, T0901317, on ovarian cancer cell proliferation and apoptosis as a potential therapeutic agent.

**Results:**

T0901317 treatment resulted in a significant (*P *<*0.001*) inhibition of cell proliferation in a time- and dose-dependent manner in CaOV3, SKOV3 and A2780 cells. Western blot analysis demonstrated an induction of p21 and p27 with a concominant reduction in phospho-RB protein levels. Cell cycle analysis demonstrated a significant (*P *<*0.001*) arrest in the G1 cell cycle phase. Significant induction of Caspase-3 and BAX gene expression occurred with treatment. Induction of apoptosis was confirmed by significant (*P < 0.001*) elevation of caspase activity on FACS analysis, caspase-glo assay, BAX protein induction and decreased caspase 3 precursor protein expression on Western blot analysis. LXR α/β knockdown experiments did not reverse the anti-proliferative and cytotoxic effects of T0901317.

**Conclusions:**

The LXR agonist, T0901317, significantly suppresses cell proliferation and induces programmed cell death in a dose- and time-dependent manner. Our results indicate that T0901317 induces its anti-proliferative and cytotoxic effects via an LXR-independent mechanism.

## Background

Ovarian cancer is the most common cause of cancer related death from gynecologic tumors and the fourth leading cause of death due to cancer in women [1,2]. The insidious nature of the disease precludes early diagnosis, therefore surgical debulking and chemotherapy are considered as standard treatment modalities for advanced stages [3]. Although the majority of patients with advanced stages of the disease respond to chemotherapy, most will ultimately succumb to the disease due to the development of chemoresistance [4]. For this reason, there is extensive research being performed searching for novel therapies to overcome chemoresistance and to develop more effective chemotherapeutic agents.

Liver X receptor-α (LXRα) and LXRβ (also known as NR1H3 and NR1H2, respectively) were discovered more than a decade ago [5]. LXRα is highly expressed in the liver and at lower levels in the adrenal glands, intestine, adipose, macrophages, lung, and kidney, whereas LXRβ is ubiquitously expressed [6]. LXR receptors and their ligands are involved in the regulation of efflux of cholesterol from atherosclerotic plaques which have led to their interest in their application for the treatment of atherosclerosis [7,8]. Synthetic LXR ligands have been developed, namely GW3965 and T0901317, and have been observed to have potential therapeutic properties in murine models for the treatment of atherosclerosis, diabetes, and Alzheimer's disease [9,10]. Over recent years, the antineoplastic properties of LXR agonists have been observed in human carcinomas such as breast and prostate, making the molecule an attractive antineoplastic agent for investigation in the treatment of ovarian cancer [11-15]. In this study we investigated the effects of a synthetic LXR agonist, T0901317, in various human ovarian cancer cell lines. LXR agonist, T0901317 may be a promising therapeutic agent in the treatment of ovarian cancer.

## Methods

### Materials

Synthetic non-steroidal LXR agonist *N*-(2,2,2-trifluoro-ethyl)-*N*-[4-(2,2,2-tri-fluoro-1-hydroxy-1-trifluoromethyl-ethyl)-phenyl]-benzene sulfonamide (T0901317) was purchased from Sigma (Saint Louis, MO). Dulbecco's Modification of Eagle's Medium (DMEM), Hank's Balanced Salt Solution (HBSS) and Fetal Bovine Serum (FBS) were purchased from Mediatech (Herndon, VA). Protease inhibitor cocktail and enhanced chemiluminescence (ECL) reagents were from Roche Applied Science (Indianapolis, IN). Vybrant FAM Caspase-3 and -7 Assay Kit (V35118, Molecular Probes, Eugene OR). Anti-p27 (sc-528, 1:200), anti-BAX (sc-7480, 1:200), anti-caspase 3 precursor (sc-7148, 1:200), anti-LXRα (sc-1202 1:200), anti LXRβ (sc-130412, 1:200) antibodies were from Santa Cruz Biotechnology (Santa Cruz, CA). Anti-p21 (ab-7960-1, 1:100), and anti-β actin (ab-8229, 1:1000) antibodies were from Abcam (Cambridge, MA). Anti-phospho Rb (Ser 807/811) (#9308, 1:1000) was from Cell Signaling Technology (Danvers, MA).

### Cell Culture

CaOV3, SKOV3, A2780 (human ovarian carcinoma cell lines) and HS-68 (human foreskin fibroblasts) cell lines were obtained from the American Type Culture Collection (Manassas, VA). CaOV3 and HS-68 cells were maintained in DMEM, and SKOV3 and A2780 cells were maintained in RPMI. Media was supplemented with 10% FBS, 10 mM Hepes buffer, 1 mM Na-pyruvate, 2 mM L-glutamine, 100 units/ml penicillin, 100 μg/ml streptomycin, and cultured at 37°C in an atmosphere of 5% CO_2 _and 95% oxygen.

### Cell Proliferation Assay

CyQuant Cell proliferation assay kit was used according to manufacturer's specifications. CaOV3, SKOV3, and A2780 cells were plated at 1 × 10^4 ^cells/well in 100 μL of cell solution in Microtest 96 tissue-culture-treated polystyrene 96-well plates (Falcon; Becton Dickinson, Franklin Lakes, NJ) at 37°C at 5% CO_2_. Cells were allowed to adhere to the plate surface for 24 h, following adherence the media was aspirated and replaced with treatment media (5, 10, 20, 40 or 50 μM of T0901317 or vehicle alone). Cells were grown under these conditions for 24 to 72 h. At indicated time points, the wells were washed with PBS and subsequently frozen at -70°C overnight. 200 μl of the CyQuant GR dye/cell-lysis buffer was added to each well and incubated for 2 to 5 minutes at room temperature, protected from light. Plates were then measured using a fluorescence microplate reader with filters at 480 nm excitation and 520 nm emission maxima.

### Western Blot Analysis

1.5 × 10^6 ^ovarian carcinoma cells were cultured as above in 100 mm dish in DMEM with above described supplements for 24 h prior to T0901317 treatment. After treatment cells were washed twice in ice-cold HBSS and were lysed in ice-cold lysis buffer (50 mM Tris-HCl, pH 7.4, 150 mM NaCl, 1% Nonidet P-40, and 0.1% SDS), supplemented with protease inhibitors (10 μg/ml leupeptin, 10 μg/ml pepstatin A, 10 μg/ml aprotinin, and 1 mM of 4-(2-aminoethyl) benzenesulfonyl fluoride). Sample protein concentrations were determined via the Biorad Protein assay strictly following the manufacturer's instructions. Proteins (30-40 μg/lane) were separated on a denaturing 8% SDS polyacrylamide gel and transferred to a nitrocellulose membrane. Membranes were blocked in 1% blocking solution in phosphate-buffered saline (PBS) and subsequently incubated overnight at 4°C with primary antibody. After washes, the membranes were incubated with secondary antibody conjugated to horse radish peroxidase for 1 h at room temperature. Chemiluminescence was detected using the ECL reagent according to the manufacturer's protocol. Different exposure times were used to ensure that bands were not saturated. For detection of β-actin, the same membranes were incubated with rabbit polyclonal anti-beta actin antibody overnight at 4°C and processed as described.

### Flow Cytometric Analysis

Aliquots of cells (1 × 10^6^/ml) were fixed in 70% ethanol for 2 hours at 4°C; cells were then centrifuged at 1500 rpm, and the resulting pellets were resuspended in 1 ml of freshly prepared propidium iodide/RNase solution. Cell cycle distribution was analyzed with the GuavaEasy Cyte mini system by using the Guava CytoSoft Cell Cycle Program according to the manufacturer's instructions (Guava Technologies, Hayward, CA). Based on the intensity of the propidium iodide fluorescence, the flow cytometry program will separate resting cells with one copy of each chromosome (G0/G1), cells that have replicated and contain double DNA content and thus double intensity of fluorescence (G2/M) and cells in S phase.

### Caspase-3 and -7 assay

Vybrant FAM Caspase-3 and -7 Assay Kit V35118, (Molecular Probes, Eugene OR) was used to quantitatively determine the percentage of cells actively undergoing apoptosis according to the manufacturer's instructions. Briefly, ovarian carcinoma cells were seeded overnight in 6 wells plates at a density of 2 × 10^5 ^per well. Cells were then treated for 24 h with T0901317 (10 μM) or 0.1% DMSO as negative control. Cells were then trypsinized and collected and 1 × 10^5 ^cells per sample were stained with 10 μl of FLICA reagent and 7-AAD and incubated at 37°C in 5% CO_2 _for one hour. Cells were then washed with 1× wash buffer, centrifuged at 1500 RPM for 5 minutes. The supernatant was discarded, 400 μL of 1× wash buffer was added and samples were analyzed by flow cytometry according to manufacturer's recommendations (Calibur, BD Biosciences).

### Caspase-3/7 activation assay

Caspase-3/7 activation assays were performed using a Caspase-Glo™ 3/7 assay kit (Promega, Madison, WI) according to the manufacturer's instructions. Briefly, ovarian carcinoma cells were seeded in 96-well plates at a density of 1 × 10^4 ^cells/well. After 24 h, cells were treated with different concentrations of T0901317 (5, 10, 20, 40 and 50 μM) or 0.1% DMSO as negative control. Caspase-Glo 3/7 reagent (100 μl) was then added to each well including medium alone, untreated control cells or cells treated with T0901317 for 6 h. The plate was then incubated at room temperature for 1 h and the luminescence of each sample was measured with a Veritas Microplate Luminometer (Turner BioSystem, Sunnyvale, CA).

### RNA Interference

Ovarian carcinoma cells were plated at a density of 1.5 × 10^5 ^cells per well in 12 well plates. Allowed to adhere for 24 hours, subsequently the cells were transfected at a confluence of 50-60% with 200 nM of validated LXR-α/LXR-β siRNA (Dharmacon, NR1H3/NR1H2) using the Mirus transfection reagent (Mirus, TransIT-TKO, MIR 2150). Cells remained with transfection complexes for 48 hours and subsequently the knockdown efficiency was assessed via real time RT-PCR.

### Real Time RT PCR

Total RNA was isolated according to recommendations by the manufacturer using the RNeasy kit (QIAGEN, Valencia CA). The RNA was quantified using the Genequant spectrophotometer and reverse transcription was performed using SuperScript II Reverse Transcriptase and reagents from Invitrogen (USA), strictly following manufacturer's instructions. Real time PCR was performed using Taqman and gene specific primer FAM probe mixes (Applied Biosystems, Foster City CA). Expression of LXR-α, LXR-β, BCL-2, BAX, Caspase-3 and beta-actin as endogenous control was analyzed. The reactions were run in triplicate in the ABI 7500 system (Applied Biosystems) and results were analyzed with SDSv1.3 software that uses the ΔΔCt method for relative quantification.

### Multitox-Glo Multiplex Cytotoxicity Assay

Cells were plated at a density of 5 × 10^3^cells/well in a 96 well plate, and allowed to adhere overnight. After T0901317 treatment, 100 μL of the fluorogenic, cell permeant reagent GF-AFC, (Promega, Madison WI) and incubated for one hour, following suggested protocol from the manufacturer. Samples were then analyzed using a Wallac Victor microplate Fluorometer.

### Data Analysis

Each experiment was conducted at least three times with consistent results. All values in the figures are expressed as mean value ± SD. The data were analyzed using student's T test with significance determined as *P *< 0.05.

## Results

### Characterization of antiproliferative effects of T0901317 treatment in CaOV3, SKOV3 and A2780 ovarian cancer cell lines

**Figure 1:**
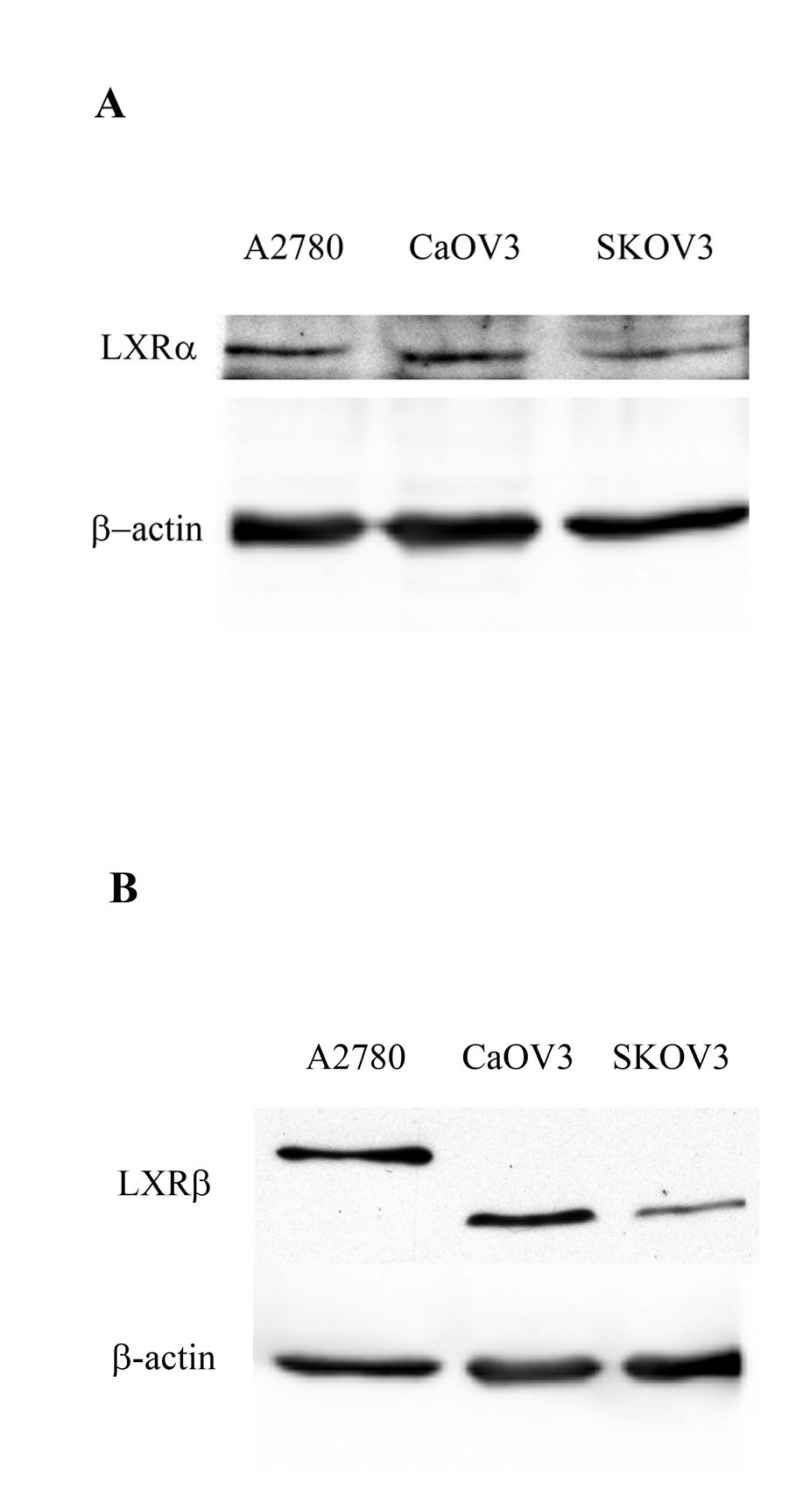
**Expression levels of LXRα/β proteins in human ovarian carcinoma cell lines**. Whole-cell lysates of A2780, CaOV3 and SKOV3 cells were obtained and subjected to immunoblotting. Forty micrograms of lysate were loaded per lane. LXRα primary antibody was used in (A) and LXRβ primary antibody was used in (B).

**Figure 2:**
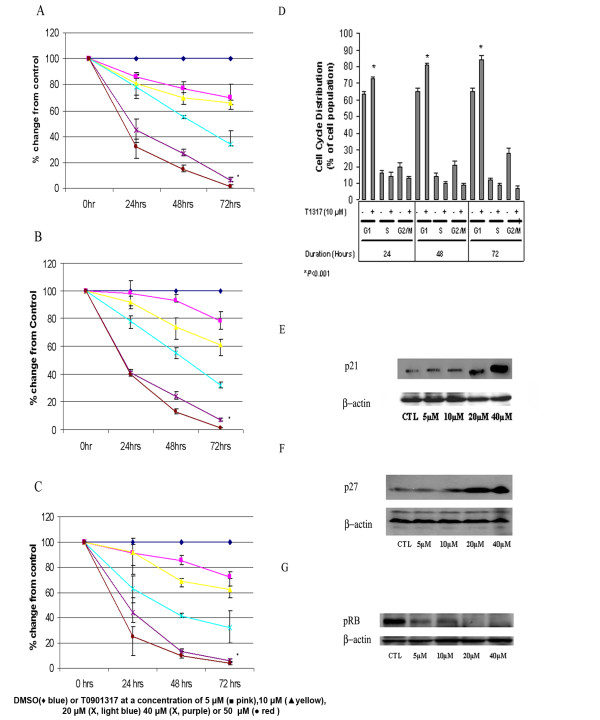
**Characterization of antiproliferative effects of T0901317 treatment in ovarian carcinoma cells**. A2780, CaOV3 and SKOV3 cells were cultured and treated with DMSO (♦ blue) or T0901317 at a concentration of 5 μM (■ pink), 10 μM (▲ yellow), 20 μM (**X**, light blue), 40 μM (**X**, purple) or 50 μM (● red) for 24 h, 48 h or 72 h (A-C). Proliferation status was determined by the CyQuant proliferation assay. T0901317 significantly inhibits cellular proliferation in all cell lines in a dose-dependent and time-dependent manner. Each value is the mean ± SD of three independent experiments, and the proliferation value is expressed as percentage of vehicle-treated cells (DMSO). (*P < 0.0001 vs. untreated cells). After culturing with vehicle (DMSO) or with T0901317 for the indicated time-points at a concentration of 10 μM, cells were stained with propidium iodide as detailed in Material and Methods and examined by flow cytometry to determine cell cycle phase distribution (D). After 24, 48 or 72 hours of treatment, the LXR agonist T0901317 decreased the percentage of cells in S phase and increased the percentage of cells in the G0/G1 phase, indicating a cell cycle arrest at the G1-S checkpoint. The percentage of cells in G0/G1 phase increases in a time-dependent manner. Results are the mean of three independent experiments and are expressed as percentage of cells, presented as mean ± SD. *P < 0.001. CaOV3 cells were grown in media supplemented with 10% FBS for 48 hours in presence of vehicle (DMSO) or the indicated concentrations of T0901317 (5 μM to 40 μM). Whole-cell extract was obtained and 60-90 μg of protein was analyzed for phospho-pRb (E), p21 (F) or p27 (G) protein levels by Western blot analysis.

The expression of LXR was studied in three commonly used ovarian cancer cell lines, A2780, CaOV3 and SKOV3, by Western blot analysis. Although the expression of LXRα protein is believed to be restricted to liver, adipose and macrophages, we observed that LXRα is constitutively expressed in ovarian carcinoma cells, as shown in Figure 1A. There was also expression of LXRβ in all three cell lines with slower migration in the A2780 cells, Figure 1B. The effects of the LXR agonist T0901317 were examined on ovarian cancer cell growth. Cells were treated with various concentrations of LXR agonist T0901317 for three days, and cellular proliferation was determined via the Cyquant cell proliferation assay. As demonstrated in Figure 2A-C, T0901317 drug treatment results in inhibition of cell growth compared to untreated cells. The effect is observed in a dose- and time-dependent manner. Drug treatment with a dose of 20 μM, on cell proliferation in all three ovarian carcinoma cell lines is similar and significant (*P < 0.001*) after a 72 hour treatment. CaOv3, SKOV3, and A2780 ovarian cancer cells demonstrated a 34% ± 9, 32% ± 4, and 32% ± 12 change in cell number compared to untreated cells, respectively. Analysis of cell cycle was performed via flow cytometry. As shown in Figure 2D, CaOV3 cells treated with 10 μM of T0901317 after 24 hours demonstrated a significant (*P < 0.001*) 9% ± 1 increase in the percentage of cells in the G0/G1 phase with a concomitant decrease in the G2/M phase (7% ± 1), compared to vehicle-treated cells. Similar results were obtained after 48 and 72 hours of T0901317 treatment with a significant (*P *<*0.001*) increase in the percentage cells in the G0/G1 phase (16% ± 2 and 19% ± 3, respectively). Percentage of cells in the S-phase had decreased at each time point, for instance from 14% ± 1 to 10% ± 2 at 48 hours. Associated decrease of cells in the G2/M phase was also demonstrated (12% ± 1 and 21% ± 3 at 48 and 72 hours, respectively). To further elucidate the mechanism through which T0901317 arrests cell cycle progression, we analyzed the expression of selected G1→S check point -proteins via Western blot analysis. Both p21 and p27 inhibit the activity of the cyclin D/CDK4, cyclin E/CDK2, cyclin A/CDK2 complexes, and the phosphorylation of pRb, resulting in G0/G1 cell arrest. As demonstrated in Figure 2E, F, treatment of CaOV3 cells with T0901317 resulted in an increase of p21 and p27 protein expression in a dose-dependent manner after 48 hours. Treatment with T0901317 resulted in a dose-dependent inhibition of Rb phosphorylation at Ser807/811, as shown in Figure 2G. Human foreskin fibroblasts (HS-68) were utilized in order to determine the effects of T0901317 on non-malignant cells. T0901317 did not cause any significant inhibition of proliferation (data not shown).

### 3.3 Morphologic changes and decreased cell density demonstrated microscopically after T0901317 treatment

**Figure 3:**
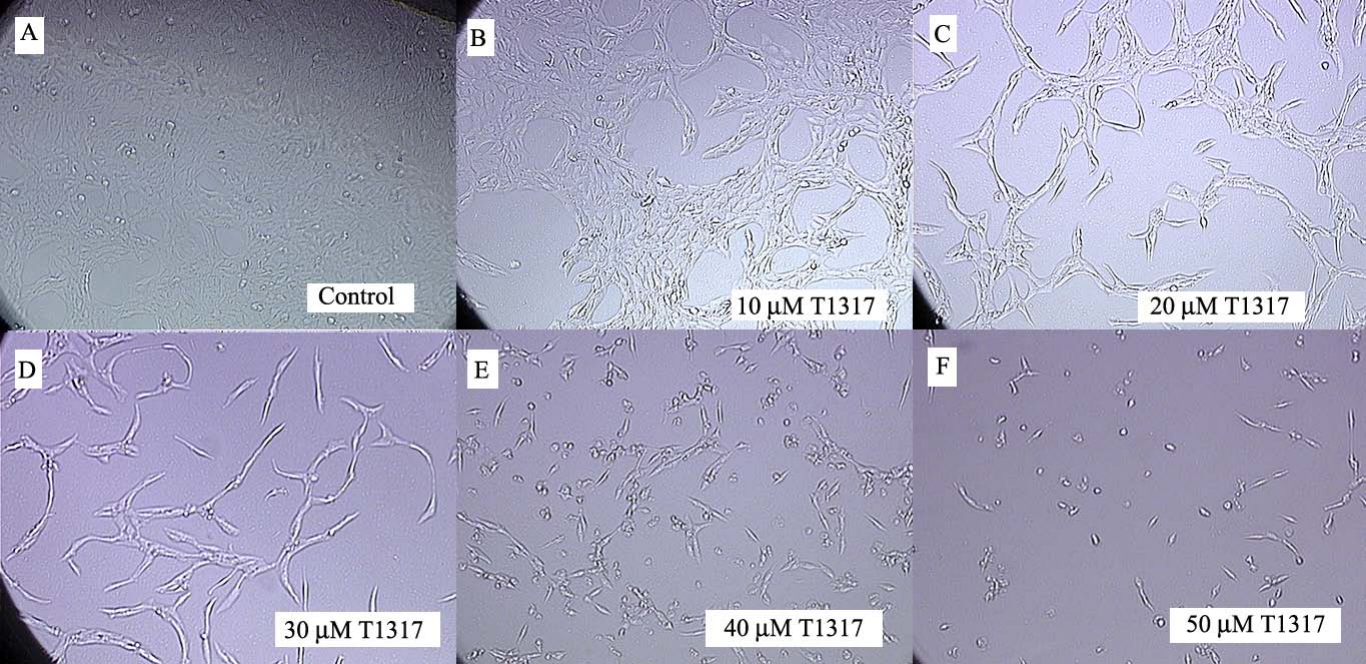
**Effect of the LXR agonist T0901317 on cellular morphology**. CaOV3 cells were cultured and treated with DMSO (1%, A) or T0901317 at a concentration of 5 μM (B), 10 μM (C), 20 μM (D), 40 μM (E) or 50 μM (F) for a total of 48 hours. Cells were visualized microscopically (10X) and pictures taken. The pictures clearly demonstrate a significant effect on cellular morphology. At increasing doses of the LXR agonist, the cells appeared to have a decreased amount of cytoplasm with a concomitant decrease in cell cumber. At the doses of 40 μM and 50 μM, the cells appeared apoptotic with necrotic debris present in the media.

As seen in Figure 3A-F, the changes are quite dramatic. The cells were photographed and viewed at 100× magnification using the Nikon TE 600 series microscope. With increasing doses of the LXR agonist, the morphologic changes included decreased cytoplasm with a spindle-like formation that appears apoptotic at the highest concentrations. Additionally, the cell density is concomitantly reduced.

### Determination of pro-apoptotic effects with T0901317 treatment

**Figure 4:**
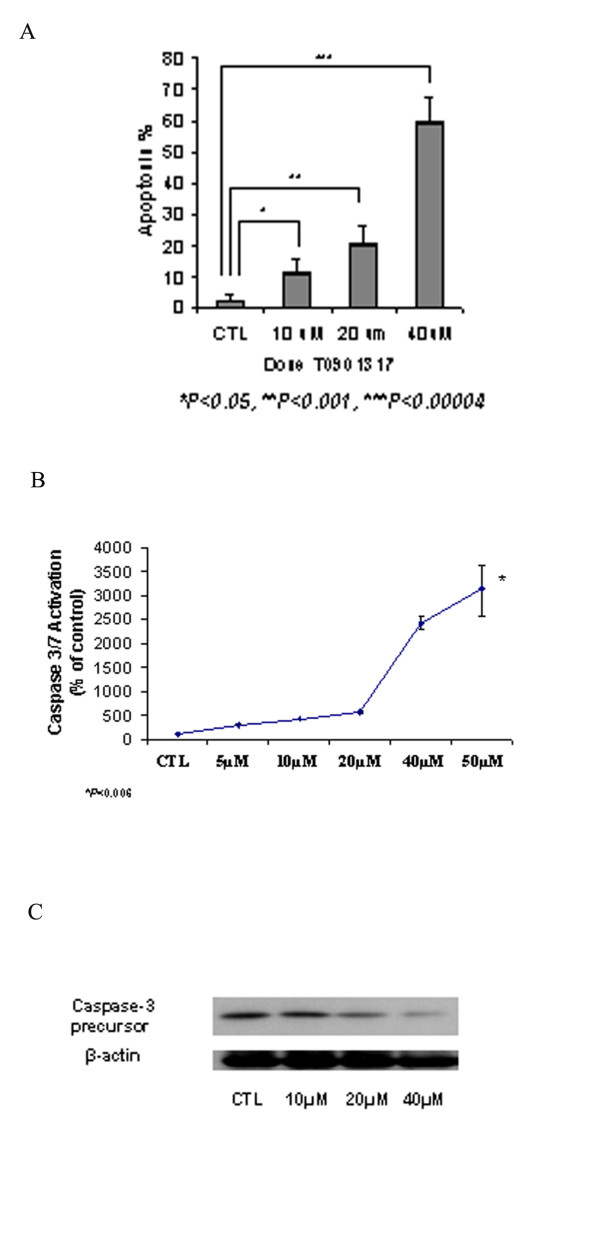
**Induction of apoptosis with T0901317 treatment**. Flow cytometric analysis of apoptosis was utilized for determination of caspase-3 and -7 activities. CaOV3 cells were treated with either vehicle (DMSO) or T0901317 at the indicated doses (10 μM to 40 μM) for 24 hours and then stained with Vybrant FAM dye, and 7-AAD strictly following manufacturer's instruction. Data are mean ± SD of three different experiments (A). Caspase 3/7 activity was also measured in CaOV3 cells after 12 hours of treatment with vehicle (DMSO) or 5 μM, 10 μM, 20 μM, 40 μM or 50 μM. A luminescent assay was used, as detailed in Material and Methods. T0901317 significantly increases Caspase 3/7 activation. Results are the mean ± SD of three independent experiments and are expressed as percentage of negative control (DMSO). (* p < 0.006 vs. negative control, (B). The activation of caspase 3 was confirmed by Western Blot analysis. LXR agonist treatment enhances caspase 3 activation, resulting in increased caspase 3 precursor cleavage rate and decreased caspase 3 precursor protein levels. Decreased caspase-3 precursor protein levels occur in a concentration dependent manner (C). β-actin expression was determined by Western blot analysis and used as an endogenous control.

**Figure 5:**
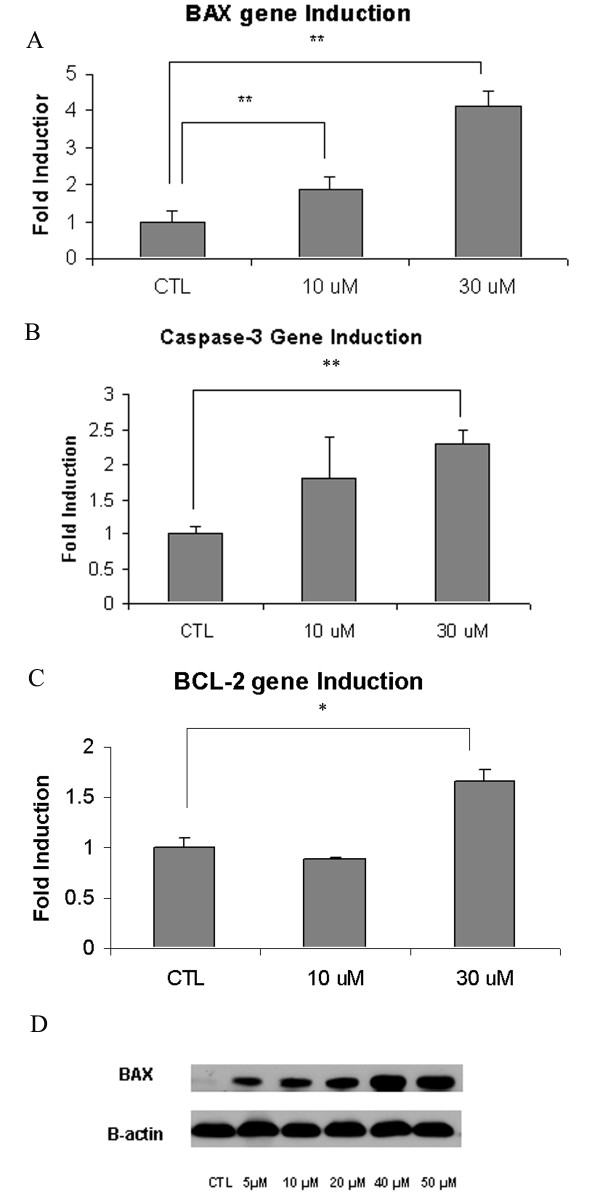
**Effect of T0901317 treatment on apoptotic gene and BAX protein expression**. After a 24 hour treatment, cells were harvested for isolation of mRNA as detailed in the methods section. A significant induction of BAX and caspase gene induction was demonstrated, especially at the 30 μM dose. Upregulation of the anti-apoptotic Bcl-2 gene expression was demonstrated with the 30 μM concentration (A-C), *P < 0.05, **P < 0.001). CaOV3 cells were grown in media supplemented with 10% FBS for 24 hours in presence of vehicle (DMSO) or the indicated concentrations of T0901317 (5 μM to 50 μM). Whole-cell extract was obtained and 60 μg of protein was analyzed for BAX protein levels by Western blot analysis. β-actin expression was used as an endogenous control (D).

We examined apoptosis in CaOV3 cells by measurement of caspase -3 and -7 activity via flow cytometric analysis. Figure 4A shows the percentage of cells in early apoptosis, as assessed by Vybrant FAM Caspase 3-and 7 Assay Kit and 7-Amino-Actinomycin D (7 AAD) staining. Treatment with T0901317 resulted in a significant (*P *< 0.05) increase of cells in early apoptosis from 2.2% ± 2 in vehicle-treated cells to 10.7% ± 5 after a 24 hour treatment (10 μM). At a higher dose of 40 μM, the cells in early apoptosis significantly (P < 0.00004) increased to 59.5% ± 8. Additionally, caspase 3 and 7 activation was measured via a luminescent assay (Caspase-Glo). We found a significant (P < 0.0006) increase in caspase 3 and 7 activity in cells treated for 24 h with the LXR agonist at a dose of 50 μM. As seen in Figure 4B, in T0901317 treated cells Caspase 3/7 activity was 287% ± 36 (5 μM), 420% ± 27 (10 μM), 580% ± 56 (20 μM), 2,406 ± 242 (40 μM) and 3,158% ± 601 (50 μM) compared to vehicle-treated cells. We confirmed caspase 3 activation by investigating the caspase 3-precursor protein level by Western blot analysis (Figure 4C). We noted a decreased level of caspase 3-precursor protein after 24 hours of T0901317 treatment. We then examined the effect of T0901317 treatment on apoptotic gene induction, and we observed a significant (*P *< 0.05) upregulation in gene expression of selected pro-apoptotic genes, specifically BAX and caspase-3, at the dose of 30 μM (Figure 5A-C). An induction of the anti-apoptotic gene, BCL-2, was also demonstrated at the 30 μM concentration. At the dose of 10 μM, a significant (P < 0.05) induction of BAX gene expression was demonstrated. After 48 hours, the level of BAX protein expression increased in a dose- dependent manner (Figure 5D).

### Attenuation of LXR-α/β expression by siRNA does not reverse the anti-proliferative effect of T1317

**Figure 6:**
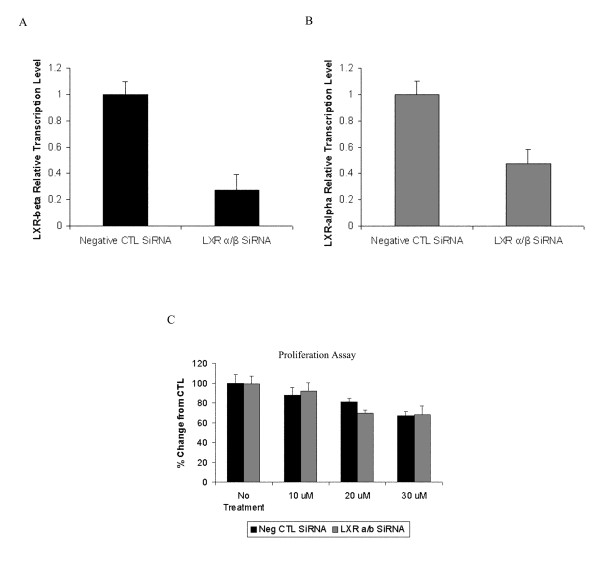
**Effect of LXRα/β inhibition on cell proliferation in T0901317 treated CaOV3 cells**. (A) Evaluation of LXRα and (B) LXRβ expression in siRNA transfected cells by quantitative real time RT-PCR. (C) siRNA transfected CaOV3 cells were cultured and treated with DMSO or 20 μM T0901317 for 24 hours. The cell growth of was assessed by the Cyquant proliferation assay. Each value is the mean ± SD of three independent experiments (* p < 0.05 vs control siRNA).

In order to determine whether the growth inhibitory effect of T0901317 is mediated by LXR, siRNA experiments in CaOV3 cells were done to decrease expression of LXRα/β and then assayed cellular proliferation in response to LXR agonist. As shown in Figure 6A and 6B, expression of LXRα was inhibited by 70% and of LXRβ by 50%. However, inhibition of LXRα/β did not prevent the anti-proliferative effect demonstrated after T0901317 treatment (Figure 6C).

### Effect of T0901317 Treatment on an FXR-dependent gene, short heterodimer partner (SHP) in ovarian carcinoma cells

**Figure 7:**
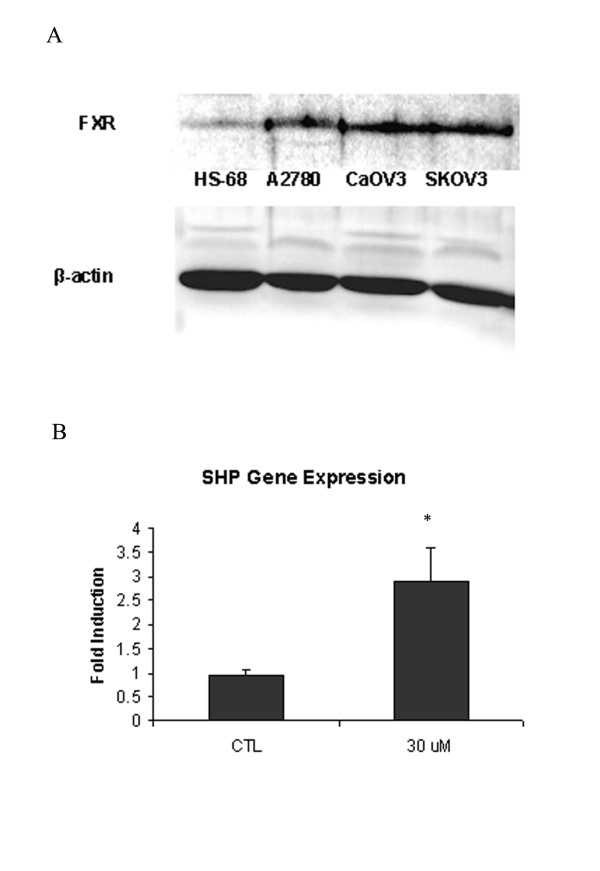
**Effect of T0901317 Treatment on an FXR-dependent gene, short heterodimer partner (SHP) in ovarian carcinoma cells**. (A) Whole-cell lysates of HS68, A2780, CaOV3 and SKOV3 cells were obtained and subjected to immunoblotting. Fifty micrograms of lysate were loaded per lane and the blot was probed with anti-FXR antibody. (B) CaOV3 cells were treated with T0901317 for 24 hours and SHP gene mRNA expression was examined by real time RT-PCR. (*P < 0.001)

The concentration used for our studies, 10 to 40 μM agonist suggests activation of alternate receptors such as the farnesoid-X receptor (FXR). The expression of FXR was evident in HS68, A2780, CaOV3, and SKOV3 cells via Western Blot analysis (Figure 7A). A 24 hour treatment with T0901317 of CaOV3 cells resulted in significant (*P *< 0.05) induction in gene expression of SHP, an FXR-dependent gene (Figure 7B).

## Discussion

Ovarian cancer has an overall poor prognosis especially in the case of chemoresistance; therefore, the development of effective chemotherapeutic agents is of ultimate importance [16]. Our study demonstrates a possible therapeutic mechanism of T0901317 which possesses anti-neoplastic properties in ovarian cancer cells with suppression of proliferation and induction of apoptosis. This is the first study to report these observations in human ovarian carcinoma cells. However, the antineoplastic properties of LXR agonists have been demonstrated in other human carcinomas such as breast and prostate [12-14]. LXRs are nuclear receptors that first were discovered to have a regulatory function in control of lipid metabolism. They were shown to have the ability to induce lipid efflux from atherosclerotic plaques [17]. Subsequently, LXR's were also demonstrated to have an additional regulatory role in immune cell function, specifically modulation of murine macrophage response to inflammatory stimuli [18].

Interestingly, our study demonstrates that the primary receptor involved in induction of cell death and cell cycle arrest is not LXR. T0901317 has been demonstrated to have agonistic effects on receptors other than LXR, such as the Pregnane X Receptor (PXR) and the Farnesoid X Receptor (FXR) [19]. According to a study by Houck, et al., the principal receptor activated at a dose of 1 μM and below, primarily activates the Liver X Receptor, whereas doses above 1 μM primarily activate the farnesoid X receptor (FXR) [20]. Interestingly, a Phase I pharmacokinetic trial and correlative *in vitro *Phase II tumor kinetic study of apomine, a FXR agonist, demonstrated inhibition of tumor growth from patients with ovarian cancer [21]. A study by Swales, et al. demonstrated the ability of an FXR agonist, GW4064, to induce apoptosis and inhibit proliferation in breast cancer cells [22]. Therefore, it is likely that FXR activation by T0901317 may lead to induction of apoptosis and cell cycle arrest in ovarian cancer cells. T0901317 has the ability to induce the gene expression of short heterodimer protein (SHP), which is involved in bile acid synthesis regulation, and is reported to be an FXR-dependent gene [23]. Despite T0901317 being a synthetic LXR agonist, the concentration dependent activation of other receptors must be taken into account when studying this compound.

We have demonstrated the effect of T0901317 on ovarian cancer cell morphology and on cellular proliferation. These occur in a time- and dose-dependent manner, which are similar to findings reported in a study by Wente, et al., describing inhibition of cell proliferation in insulinoma cells [15]. Cell cycle analysis indicated that T0901317 induced G0/G1 cell cycle arrest with a concomitant decrease in both the S and G/M2 phases. A study in human prostate cells demonstrated similar findings with a decrease in the percentage of cells in the S-phase after treatment [13]. We analyzed the expression of p21 and p27 which are regulatory proteins involved in G0/G1 phase arrest, via inhibition of cyclin/CDK complexes that are necessary for cell cycle progression [24]. One such mechanism for cell cycle progression into the S-phase is phosphorylation of the retinoblastoma (Rb) protein by cyclin/CDK complexes [25]. Our study demonstrates that upregulation of both p21 and p27 correlates with inhibition of phosphorylation of the Rb protein, therefore causing G0/G1 cell cycle arrest and inhibition of cellular proliferation.

We analyzed the ability of T0901317 to induce apoptosis in ovarian cancer cells. T0901317 has a significant ability to induce the activity of caspase-3 and -7 leading to apoptosis in ovarian carcinoma cells. Further evidence is elucidated by the induction of caspase-3 and BAX gene expression. Induction of the pro-apoptotic protein, BAX, was upregulated in a dose-dependent manner. The BAX protein is a member of the Bcl-2 family, and when over expressed has the ability to accelerate apoptosis [26].

## Conclusion

To our knowledge, this is the first study to report the anti-proliferative and pro-apoptotic activity of T0901317 on ovarian cancer cells mediated via an LXR-independent pathway. We believe that based on our results that synthetic LXR agonists warrant further studies as anti-neoplastic agents in the treatment of ovarian cancer.

## Conflicts of interests

The authors declare that they have no competing interests.

## Authors' contributions

JR carried out proliferation/apoptosis assays, knockdown experiments along with drafting of the manuscript. SY carried out Western Blot and flow cytometry analysis. MAM assisted in conception of study and aided in the drafting of the manuscript. JMD coordinated the study and provided funding of the studies. All authors read and approved the final manuscript.
